# Women’s Views and Experiences of the Triggers for Onset of Bacterial Vaginosis and Exacerbating Factors Associated with Recurrence

**DOI:** 10.1371/journal.pone.0150272

**Published:** 2016-03-01

**Authors:** Jade Bilardi, Sandra Walker, Julie Mooney-Somers, Meredith Temple-Smith, Ruth McNair, Clare Bellhouse, Christopher Fairley, Marcus Chen, Catriona Bradshaw

**Affiliations:** 1 Central Clinical School, Monash University, Melbourne, Victoria, Australia; 2 Department of General Practice, The University of Melbourne, Melbourne, Victoria, Australia; 3 Melbourne Sexual Health Centre, Alfred Health, Melbourne, Victoria, Australia; 4 Centre for Values, Ethics and the Law in Medicine, The University of Sydney, Sydney, New South Wales, Australia; 5 Melbourne School of Population and Global Health, The University of Melbourne, Melbourne, Victoria, Australia; University of Illinois at Urbana-Champaign, UNITED STATES

## Abstract

**Background:**

Bacterial vaginosis (BV) is the most common vaginal infection affecting women of childbearing age. While the aetiology and transmissibility of BV remain unclear, there is strong evidence to suggest an association between BV and sexual activity. This study explored women’s views and experiences of the triggers for BV onset and factors associated with recurrence.

**Methods:**

A descriptive, social constructionist approach was chosen as the framework for the study. Thirty five women of varying sexual orientation who had experienced recurrent BV in the past five years took part in semi-structured interviews.

**Results:**

The majority of women predominantly reported sexual contact triggered the onset of BV and sexual and non-sexual factors precipitated recurrence. Recurrence was most commonly referred to in terms of a ‘flare-up’ of symptoms. The majority of women did not think BV was a sexually transmitted infection however many reported being informed this by their clinician. Single women who attributed BV onset to sex with casual partners were most likely to display self-blame tendencies and to consider changing their future sexual behaviour. Women who have sex with women (WSW) were more inclined to believe their partner was responsible for the transmission of or reinfection with BV and seek partner treatment or change their sexual practices.

**Conclusion:**

Findings from this study strongly suggest women believe that BV onset is associated with sexual activity, concurring with epidemiological data which increasingly suggest BV may be sexually transmitted. Exacerbating factors associated with recurrence were largely heterogeneous and may reflect the fact it is difficult to determine whether recurrence is due to persistent BV or a new infection in women. There was however evidence to suggest possible transmission and reinfection among WSW, reinforcing the need for new approaches to treatment and management strategies including male and female partner treatment trials.

## Introduction

Bacterial vaginosis (BV) is a highly prevalent vaginal condition affecting women of reproductive age and has been associated with serious sequelae including miscarriage, preterm delivery and increased risk of sexually transmitted infections (STIs) and human immunodeficiency virus (HIV) [1–5]. In developed nations, prevalence estimates have ranged between 10–30% among women who have sex with men and between 25–50% among women who have sex with women (WSW) [6–12]. Characterized by an imbalance in the vaginal flora, symptoms of BV include vaginal malodour—often likened to a ‘fishy odour’—and a typical white homogenous discharge [13, 14], both of which have been found to be highly distressing and embarrassing to women, impacting significantly on their self-esteem and sexual relationships [15].

Despite being the most common vaginal infection among women, the aetiology of BV remains unknown and clinical treatment options suboptimal, with past studies showing rates of recurrence in excess of 50% in 6–12 months following treatment [16]. While the causative agents and transmission of BV remains unclear, there is strong evidence of an association between BV and sexual activity [7, 15, 17, 18]. Epidemiological studies have shown BV to be associated with sexual risk behaviours that typify STIs including new or multiple sexual partners, symptomatic female sexual partners, inconsistent or lack of condom use and penile-vaginal sex [7, 17, 19–22]. High frequency of sex [23] and young age of first sex [6, 24] have also been associated with an increased risk of BV. In a study of 17 to 21 year old female university students in Australia, researchers found BV was absent in women with no history of sexual activity, uncommon in women with a history of non-coital activity only and associated with penile-vaginal sex [18]. Consistent condom use and male circumcision have also been shown to be associated with a reduced risk of BV [25, 26], and while there is no clinical correlate of BV in men, many of the BV-associated bacteria have been detected in the coronal sulcus and distal urethra in males and detection has been associated with sexual activity and lack of circumcision [27]. Epidemiological studies have also found a number of ethnic, lifestyle and non-sexual behavioural factors including douching, smoking, stress and non-white ethnicity to be associated with BV [19, 28–31] however whether these are causally associated or confounders is not clear.

Despite the emerging epidemiological evidence, the difficulties in establishing the aetiology of BV and the failure of a number of male partner treatment trials to reduce recurrence in women [32, 33], it is not clear if sexual transmission of BV is occurring. Current international guidelines therefore do not characterise BV as an STI and do not recommend partner treatment [34, 35], hence women are informed by their treating clinicians that BV is not sexually transmitted.

The aim of this study was to explore women’s views and experiences of the triggers for BV onset and exacerbating factors associated with recurrence. While past BV studies have largely focused on prevalence and risk factors associated with BV, there are very limited data on women’s views and experience of first and recurrent episodes of BV.

## Methods

Detailed methods for this study have been outlined in an earlier paper [15]. This study has been reported in accordance with the Consolidated criteria for reporting qualitative research (COREQ) guidelines [36].

### Ethics statement

Ethical approval for this study was granted by the Alfred Hospital Ethics Committee, Victoria, Australia, Application Number 318/12 on the 23^rd^ October 2012.

### Synopsis of Methods

A social constructionist approach was chosen as the framework for the study. Semi-structured interviews were chosen to allow women the opportunity to tell their lived experiences and personal realities of recurrent BV while also allowing for the exploration of key clinical areas of interest. Women 18 to 45 years, who had experienced two or more diagnosed episodes of BV in the past five years and had a good understanding of verbal and written English were eligible for the study. Women were purposively sampled to allow for a broad sample of women including heterosexual and WSW (including women identifying as bi-sexual, lesbian, queer, pansexual and transgender), single women and women in a relationship, women who had experienced high and low numbers of recurrent BV and women from a number of recruitment locations. Women were recruited from the Melbourne Sexual Health Centre (MSHC), the largest sexual health clinic in Victoria, Australia; a previous longitudinal BV study of Australian WSW (The WOW Health Study) [19]; and specialist sexual health medical clinics or general practices with a high case load of female patients of reproductive age (hereafter referred to as high caseload clinics). Participants had the option of being interviewed either by telephone or face to face at MSHC or in their own home. Participants interviewed face to face at MSHC were provided with a written plain language statement (PLS) and consent form to read and sign. Participants interviewed by telephone were read aloud the PLS and consent form and asked to provide verbal consent. Verbal consent was obtained for telephone interviews as it was not practical to obtain written consent for this method of interview. Verbal consent was recorded on the consent form by way of the researcher signing on the participant’s behalf and a copy forwarded by post for their records. This process of written and verbal consent was approved by the Alfred Hospital Ethics Committee.

After obtaining informed consent, women were asked a series of 15 structured demographic, sexual behaviour and diagnosis and treatment questions before being asked open ended questions pertaining to their knowledge of BV prior to their first episode, their first and recurrent experiences of BV, the impact of BV on them emotionally, socially, sexually and in their work lives, their beliefs around the causes and triggers of BV, their use of self-help remedies and their experience of antibiotic treatment and the clinical management of BV. Findings relating to the impact of BV on women emotionally, socially, sexually and in their work lives are reported in a previous paper [15] and findings around women’s management of recurrent BV and their experiences of clinical care are presented in an upcoming paper. All interviews were conducted by JB or SW between November 2012 and January 2013. All data was collected in a once-off interview and no women required re-interviewing. Thematic analysis [37] was undertaken and data coded using primarily a segmented approach [38]. Transcripts were imported into N-Vivo 9 for data management and a subset of transcripts reviewed independently by two other research team members to cross check coding and themes (MTS, SW). Analyses of demographic, sexual behaviour and diagnosis and treatment data were conducted using SPSS 20.0.

## Results

Of the 40 women who were referred to or registered their interest in the study, 35 completed an interview, 3 were ineligible after eligibility was checked again prior to organising an interview and 2 could not be contacted. Contact details only were collected on women who did not participate. Participant demographics are shown in Table 1. The psychosocial impacts of recurrent BV have been reported in a previous paper [15].

**Table 1:** Recruitment site and participant characteristics (demographic, sexual behaviour, diagnosis and symptoms of BV) N = 35.

	N orMedian [Range]
**Recruitment site**	
MSHC	22
Longitudinal BV study	7
High caseload clinic	6
**Age**	30 [21–43]
**Born in Australia**	21
**Education level**	
Secondary school	5
TAFE diploma or certificate	9
Undergraduate degree	14
Post graduate certificate or degree	7
**Employment status**	
Full time	11
Part time	7
Casual	2
Student/Student & part time work	11
Unemployed	4
**Sexual Identity**	
Heterosexual	19
Lesbian	7
Queer	3
Bisexual	4
Other (pansexual/transgender)	2
**Sex industry worker^**	
No	29
Yes	6
**Smoke cigarettes**	
No	23
Yes	10
Past smoker	2
**Regular relationship**	
No	14
Yes	21
**Sex of partner**	
Male	13
Female	8
**Number of male sexual partners <5 years (if > = 1)**	10 [1–1300]
**Number of female sexual partners <5 years (if > = 1)**	3 [1–45]
**Number of times had BV in the past**	4 [2–35]
**Number of times had BV diagnosed in the past**	3 [2–25]
Symptoms*	
Abnormal odour	34
Abnormal discharge	35
**Most distressing symptom**	
Abnormal odour	30
Abnormal discharge	7

### Triggers for BV onset and exacerbating factors associated with recurrence

#### BV Onset

The majority of women predominantly reported sexual triggers for the onset of BV and both sexual and non-sexual factors associated with recurrence. ‘BV onset’ refers to the triggers women reported preceding and temporally associated with their first episode of BV or a further ‘new episode’ of BV. ‘Exacerbating factors’ refers to the factors women perceived as being associated with the exacerbation of recurrent symptoms of BV. This study did not investigate whether women’s BV recurrences were indicative of new infection or persistent infection.

The five main sexual triggers for BV onset identified by women were sex with a regular female partner, sex with a regular male partner, sex with a new male or female partner, sex with an uncircumcised male partner and condom-less sex with a male partner. Only a minority of women did not attribute BV onset to sexual contact either because they had experienced episodes during times they had not been sexually active, they did not have a clear sense of what triggered disease onset and/or factors associated with recurrent symptoms, or they felt other non-sexual lifestyle factors—stress, the termination of pregnancy or the use of an intra-uterine device—were responsible for BV onset. Table 2 provides example quotes of the factors women think triggered BV onset.

**Table 2:** Triggers for BV onset.

**Sexual triggers for BV onset**	
**Regular female partner**	*I was with her for 5 or more years and never had anything*, *either of us*. *And then…after we broke up*, *she then slept with another person and we then had sex again*, *the 2 of us*, *and I think*, *I started experiencing the discharge and odour after I had sex with my ex again… I mean there’s a pretty obvious link with my partner having it and then that we both had it at the same time… (Participant 20*, *age 23)*.* *.* *.* *.*the partner that I’m with now*, *I’ve been with her for almost four years*, *and then we both sort of noticed that we got it around the same time*, *a similar time*. *Not long after we first started being sexually active*.* *.* *. *(Participant 4*, *age 40)*.
**Regular male partner**	*…It’s since I met my husband*. *I met him 5 years ago and it just seems to be recurrent since then (Participant 25*, *age 37)*..*it was just one*, *one guy who I was in a relationship with*.* *.* *.*I’d never had a problem before until this one particular occasion and then since then*.* *.* *.*it hasn’t really gone away (Participant 15*, *age 37)*.
**New male or female partner**	*…it especially [happens] when I have a new partner*, *I get it frequently in the beginning and then you know it dissipated a bit…So that’s really put me off one night stands (Participant 34*, *age 42)*.*The first time that I noticed that I had something*, *I’d had a regular partner for probably about 4½ years which then stopped and I started having different partners and that was when I first sort of picked up that something had changed*.* *.* *. *when I changed partners it was almost immediate (Participant 21*, *age 27)*
**Uncircumcised male partner and/or poor sexual hygiene**	*It was my new boyfriend and not only being uncircumcised*, *also having a bit of a smell issue*.* *.* *.*I don’t have any proof*, *but I can’t help to feel that that might be it (Participant 17*, *age 34)I think at the time I felt like it was a hygiene issue with my male partner*.* *.* *.*one thing I would say is that prior to my first contraction [of BV] I probably did not have very much sex with male partners who were uncircumcised so yeah (Participant 22*, *age 28)*.
**Condom-less sex with a male partner**	*I had been with a partner for a little while and the last time we slept together the condom broke and then a few days later I started getting the symptoms (Participant 18*, *age 22)*.* *.* *.* *. *the first time I got it*, *I always suspected and it’s probably not true*, *I had a male partner who then went on to have another female partner*, *and we were then sexually active again*, *just one more time with no condoms and that was the first time I got BV and I always wondered*, *‘Did he bring back something from his other female partner*?*’ (Participant 31*, *age 34)*.
**Non-sexual triggers for BV onset**	
**Stress**	*In 2009 I broke up with my a partner*, *my male partner of 8 years because I had figured out that I was a lesbian*, *so it was a really stressful time and I think in my experience it was directly related to the amount of stress that I was under*. *So I had it twice*.* *.* *. *since then I haven’t had it again*.* *.* *.*(Participant 12*, *age 30)*.
**IUD or termination of pregnancy**	*I actually feel that it’s been more obvious since I had a termination last April*. *It has been really strong since then*. *But my doctor tells me I’ve had BV before then so she doesn’t feel that there’s a connection but I actually believe that there is (Participant 32*, *age 43)*. *I think it might have been the Mirena coil*, *like something foreign constantly inside me (Participant 26*, *age 24)*.

Interestingly, a few women reported BV onset following sex with a new partner however did not overtly make an association between BV onset and sexual contact with that partner.

Cos I had, I slept with this guy without protection, which was stupid so went to the sexual health clinic and she [the doctor] was like ‘Alright we’ll just run you through a whole lot of tests’…And then she said ‘No, it’s not actually thrush, it’s BV…’ (Participant 13, age 22).

In general, sex workers were less certain about their triggers for disease onset and did not attribute BV onset to work related sexual activity. Only one of the six sex workers felt BV onset may have been triggered by digital sex with clients, while another reported it had occurred as a result of sex with an uncircumcised personal male partner. Another woman speculated her first episode of BV may have been triggered through ‘careless sex’ with her romantic male sexual partner. The remaining three sex workers could not identify any triggers for BV onset.

I was a working girl, nothing different, like to what I usually do, it just occurred (Participant 30, age 31).

#### WSW and BV onset

WSW were more likely than heterosexual women to strongly feel their current or previous female sexual partner was responsible for the transmission of BV. Of 16 WSW in the study, over half reported they experienced BV onset or a new episode of BV following sex with a new female partner or sex with an untreated female partner with BV. The remaining women either reported BV onset following sexual contact with a male partner or were unsure of the trigger for BV onset. Of the four WSW who identified as bisexual, two reported BV onset following sex with a new male partner and new episodes with new female partners. Both women suspected their previous partners had been responsible for the transmission of BV. Of the remaining two bisexual women, one attributed BV onset to a female sexual partner and the other was unsure of the trigger for BV onset. Among WSW, a couple reported their previous partner had BV when they were first diagnosed or their most recent or current partner had since been diagnosed with BV.

…my partner had it…then I started to experience an odour and a bit of a discharge…so I asked her what was going on and she kind of told me what she thought it was and I went to a sexual health clinic just to double check and when I went the doctor diagnosed me with it [BV]. . . . (Participant 34, age 42).

…my current partner does [have BV] and my previous long term partner [did], it was the first time that I’d noticed, been around it, so some part of me wondered if I maybe got it from her? (Participant 14, age 40).

#### Exacerbating factors associated with recurrence of symptoms

Women commonly spoke of recurrences in terms of a ‘flare-up’ of symptoms, reporting a range of sexual and non-sexual factors seemed to exacerbate symptoms of BV. The five main non-sexual exacerbating factors were menstruation or the use of sanitary products, exercise, tight clothing, stress or a poor immune system. Menstruation was the most often cited exacerbating factor. To a lesser extent, women also mentioned various sexual behaviours such as frequent sex, unprotected sex or oral sex, the use of lubricant or latex based products such as condoms or gloves could exacerbate symptoms of BV.

Among WSW a couple of women strongly felt their partner had been responsible for the transmission and recurrence of BV, reporting they had experienced no further episodes since their female partner had been treated for BV or they had broken up with that particular partner.

I wondered then why I kept getting it back! It’s because my partner hadn’t been getting treated for it. Once she was treated it was fine (Participant 4, age 40).

…another friend of mine again only had BV when she was with one particular partner so it’s just, it’s just my hypothesis (Participant 2, age 39).

A couple of other WSW said they continued to experience recurrences often at the same time as their ongoing partner.

Yes we do tend to get it at the same time which is why we refrain from oral sex or any sharing of, not that we ever share toys anyway, but yes, we do notice it (Participant 3, age 39).

Table 3 provides example quotes of the exacerbating factors associated with the recurrence of symptoms.

**Table 3:** Exacerbating factors associated with recurrence of symptoms.

**Non-sexual exacerbating factors**	
**Exercise or tight clothing**	*I’ve noticed*, *you know*, *occasionally if you do a lot of exercise*, *you wear tight clothes*, *that brings it on (Participant 3*, *age 39)*.
**Menstrual cycle/ Sanitary products**	*I also tend to notice it copping up when I use tampons*, *that’s a big one for me*, *I’m on the pill and I try not to have my period at all because I find that straight afterwards something is always wrong (Participant 21*, *age 27)*..* *.* *.*it was really strong and offensive when I have my period but that’s the only time it’s really strong and offensive*, *otherwise pretty mild (Participant 31*, *age 34)*.
**Stress**	.* *.* *. *I think I’ve been going along alright and then it would be quite sudden symptoms would reappear after*, *after stressful situations*. *I’ve notice that several times since it first began*. *Really sudden*, *significant reoccurrence of symptoms (Participant 7*, *age 39)*.
**Poor immune system**	*It felt very much associated with my immune system as well*, *but yeah again it’s hard to tell (Participant 1*, *age 25)*. *I feel like in stressful*, *emotionally intense situations there must*, *my theory anyway*, *is that it impacts on my immune system and that changes my flora (Participant 7*, *age 39)*.
**Sexual exacerbating factors**	
**Oral sex**	*…usually oral sex makes it worse*, *a lot worse (Participant 30*, *age 31)*
**Frequent sex**	*Its linked to sex so the more sex I have the more frequent I seem to get it (Participant 25*, *age 37)*.
**Condom-less sex with a male partner**	*I have figured out that if I have unprotected sex it will come instantly pretty much (Participant 18*, *age 22)*. *I seem to be more conscious of unprotected sex because that seems to trigger it (Participant 24*, *age 22)*
**Lubricant**	*I have to be really careful about what lubricant I use*. *Yeah*, *I’ve found a lot of them …irritate it (Participant 28*, *age 29)*.
**Latex barriers**	*…if I’m using gloves or condoms on toys is when it seems to happen*. *So it’s actually when I use protection (Participant 34*, *age 42)*.*… and finding that condoms were sort of irritating it (Participant 22*, *age 28)*.

### Is BV an STI?

When asked directly by the interviewer, the majority of women reported they did not think BV was an STI even if they felt BV resulted from sexual contact or they felt a sexual partner was responsible for transmission or reinfection.

Not necessarily sexually contracted but definitely her flora affected mine (Participant 6, age 31).

I don’t think he would transmit BV to me. I think he might carry BV… (Participant 17, age 34).

Most women were informed by their doctors or via their own research that BV was not an STI and reported receiving varying and inconsistent advice around why BV occurs including: its ‘*just a woman’s thing’*, is associated with how sexually active you are, is an imbalance of bacteria, it could be transmitted by uncircumcised men, is similar to thrush, may be triggered by oral sex or a change in sexual partner, could be caused by soap based products or related to a low immune system.

They explained to me what it was, that it’s not an STI that sort of thing (Participant 23, age 24).

I said to the doctor today, ‘How did I get it? How did this happen?’ And he couldn’t really tell me. He said it’s not an STI but I guess I’m just curious (Participant 30, age 31)

I’m also aware that BV isn’t like an STD, it isn’t a specific germ that is passed on by your partner (Participant 24, age 22)

While most women reported they did not think BV was an STI, an interesting ambiguity was evident in the language many women used to refer to their acquisition of BV–‘*I got it*’, ‘*my first contraction*’, ‘*passed onto me*’.

### Responsibility for acquisition of BV

It was common for women to feel a sense of shame around their BV recurrences due to the embarrassment they felt about their symptoms and the social stigma associated with the symptoms of vaginal conditions and more broadly STIs.

…it’s on this weird borderline about whether it is or it isn’t a sexually transmitted disease which is just like another weird barrier for women accessing information about it because of the shame associated with having an STD…we don’t have stigma associated with getting the flu but STD’s have rolled over onto BV and that’s something to think about (Participant 28, age 29).

While most women felt embarrassed about their symptoms of BV—which impacted on various aspects of their lives [15]—some women were more likely to blame themselves for acquisition than others. Table 4 provides examples of women’s attribution of blame for BV acquisition.

**Table 4:** Attribution of blame for BV.

**Self-blame**	
**Poor sexual behaviour choices**	*…when something like this happens I tend to think ‘Well*, *if you hadn’t had sex with that person’*, *and all of the probably shame and guilt and all my own*, *my own feelings surrounding…sex and the choice of person*.* *.* *. *it still comes with a sense of ‘Well if you hadn’t done that then you wouldn’t be in this situation*!*’ (Participant 11*, *age 32)*.. . .*for that one particular time when I didn’t use any protection and it was kind of like*.* *.* *.*kind of reinforced the fact that you need to be really careful about engaging in sexual behaviour with other people (Participant 16*, *age 27)*
**Poor health and wellbeing**	.* *.* *. *it’s just that feeling of you know*, *[of] not being well and you know*, *feeling like you need to look after yourself better or something (Participant 2*, *age 39)*. *Well*, *yeah*, *it is from me reflecting on my lifestyle*, *because I do drink a lot*, *and I do nothing physical*, *so then I think*, *maybe if it’s reoccurring so much*, *then it might have something to do with my lifestyle and everything*, *so yeah*, *my whole diet’s changed (Participant 18*, *age 22)*.
**‘*What have I done*.* *.* *.?**	*So just really confused so everything was running through my head*, *is it the mirena coil*? *Has my boyfriend cheated on me and given me something*? *Have I done something wrong*? *Have I lost a tampon up there*?.* *.* *. *Did I change my soap*? *Like confused*. *I was just like ‘What have I done to do this*?*’ (Participant 26*, *age 24)*.
**No self-blame**	
**‘Just one of those things’**	.* *.* *.*it’s not your fault and it’s just a normal thing that*, *you know*, *a bit of an imbalance and you can correct it and it’s not a big deal (Participant 13*, *age 22)*. *It’s a thing that happens*, *it is*, *I’m not going to say ‘normal’*, *but a lot of people do get the same thing (Participant 5*, *age 38)*

Single women who felt BV had resulted from sex with a casual partner or unprotected sex were more likely to display stronger feelings of self-blame than women in relationships or women who attributed BV to ‘*just one of those things’* or other non-sexual lifestyle factors. These women tended to feel it was ‘their fault’ they had acquired BV due to poor sexual behaviour choices. A few single women and women in relationships who felt that poor health and wellbeing choices might be contributing factors to their recurring BV also showed a degree of self-blame, however to a far lesser extent.

In comparison, women in relationships tended not to blame themselves or their partners for BV acquisition, even though many felt certain their partner had been responsible for transmission or reinfection. WSW in particular were very open in their discussions and attempts with partners to prevent further recurrences, particularly if their partner had also experienced BV.

…well we said ‘OK we won’t have sex again to see what happens’. And we tried that and then we got it back again. So we were sort of trying different thing just ourselves too! (Participant 4, age 40)

Single women and women in a relationship who tended to attribute their BV to ‘*just one of those things*’- generally based on the advice from their clinicians—were also less likely to display self-blame tendencies. While these women commonly felt their BV was triggered by sexual contact, they were more inclined to view it as a by-product of sexual contact rather than as a consequence of their sexual or lifestyle behaviours or choices. Sex workers were also less inclined to blame themselves for the acquisition of BV even if they felt sexual contact may have been a contributing factor.

…it’s just a kind of thing that happens and… I’m not of this position where I’m generally, I guess, above par with sexual shame (Participant 22, age 28).

Interestingly, for many women, being told by clinicians that BV was not an STI or ‘*just one of those women’s things’* seemed to bring a great sense of relief and eased their tendency toward self-blame.

Oh well I used to sleep with lots of people once, and it’s sort of like ‘Well is this connected to those sort of things and stuff?’ ‘Do I have some other like disease or something?’ But now that I just know it’s this, it’s more like, ‘Ah it’s this, oh well, who cares’ (Participant 1, 25 years).

For others however, being informed BV was not an STI just left them with a sense of confusion—and still for some a lingering sense of shame—particularly if in their experience their recurrences were triggered or exacerbated by sexual contact or certain sexual behaviours.

..he [doctor] was checking it and telling me that it’s not something bad, it’s not contagious, it’s just something normal…so I was obviously feeling a lot better and knowing that I would have not done anything by having sex with people but still feeling a lot of shame around it (Participant 33, age 28).

While most women may not have overtly blamed themselves for BV acquisition, many women still questioned if they were doing something ‘*wrong*’ to keep getting BV (see Table 4) with a number of women, interestingly, equating STI acquisition with erroneous or immoral behaviour.

Like if you had it and then you don’t get it again until you do something naughty (laughs). Like I don’t feel like I’m misbehaving…like if it was an STD that was caused by having unprotected sex then with someone who is infected with something then I feel I have to deal with it, that’s my bad… (Participant 20, age 23).

### Differences between Groups of Women

As part of the study we explored possible differences in the experiences of heterosexual women and WSW, single women and women in a relationship and sex industry workers and non-sex industry workers. Differences have been reported previously [15] however in relation to the data presented in this paper, we found that sex workers were generally less certain of their triggers for disease onset and less likely to display self-blame tendencies or question their role in acquisition. As reported earlier, single women were more prone to self-blame than women in relationships and WSW were most likely to be certain of their triggers for disease onset and/or recurrences, with many attributing it to sexual contact with a new female partner or a female partner with BV. There were no major differences in the experiences of bi-sexual women and women identifying as lesbian, queer, transgender or pansexual, other than bisexual women were more likely to report first episode BV was triggered by a male sexual partner.

## Discussion

The findings from our study on women’s views and experiences of the triggers for BV onset and exacerbating factors associated with recurrence concur with epidemiological data which increasingly suggest sexual contact is associated with the development of BV. The majority of women in this study predominantly reported sexual triggers for the onset of BV, including sex with a regular female partner, sex with a regular male partner, sex with a new male or female partner, sex with an uncircumcised male partner and condom-less sex with a male partner, factors which have shown to be associated with an increased risk of BV [7, 17, 19, 20, 22, 23, 25]. Importantly, over half of WSW reported BV onset following sex with a new female partner or a female partner with BV, with a number firmly believing their partner was responsible for transmission. These data are consistent with recent findings from a cohort study of Australian WSW which found women with a new female sexual partner or female partner with BV are significantly more likely to have BV [39].

Exacerbating factors attributed to recurrence were less clear and included a range of sexual and non-sexual factors, reflecting the likely heterogeneous nature of recurrence which may reflect a new episode of infection in some cases, or a re-emergence of symptoms from persistent infection in others. While most women spoke of recurrence in terms of an exacerbation or a ‘flare up’ of symptoms—the most common factor being menstruation—a number of WSW spoke of recurrences in terms of possible reinfection from partners with a number of women reporting they no longer experienced recurrences following partner treatment or breaking up with their partner or continued to experience recurrences often at the same time as their partner. This finding is also consistent with past data which have found women who are with the same partner pre and post treatment are 2–3 times more likely to experience BV recurrence [16, 40].

Interestingly, despite the majority of women reporting BV onset following sexual contact most women did not believe BV was an STI even if they strongly felt their partner was responsible for the transmission of BV. Women’s opinions on whether BV is an STI is likely to be strongly influenced by publicly available information and advice provided to them by clinicians with many women stating they were explicitly informed by clinicians BV was not an STI. Clinicians are guided by current treatment guidelines for the management of BV, which do not report BV as an STI, nor recommend male or female partner treatment [34, 35]. It is not surprising women feel confused and unsure about the nature of BV when in their experience sexual contact seems to have triggered BV or recurrences and yet they are informed it is not an STI. Much of the language from women in this study suggests many possibly do suspect BV is sexually ‘contracted’ or ‘transmitted’ by a partner however they do not label it as such due to trusted information they have been provided or because they do not want to think of it as an STI.

While not currently termed an STI, BVs sexual, recurring nature means it is not uncommon for women to feel a similar sense of shame and stigma as that associated with an STI diagnosis [15, 41, 42]. This results from the stigma around the type of people who contract STIs–women who are deviant, dirty, immoral and sexually promiscuous [43–48]–which often leads to feelings of shame and guilt when women contract an STI themselves [43]. It was interesting to see the number of women in this study who echoed these sentiments equating STI acquisition with having done something ‘*below board*’ or ‘*naughty*’. Shame in turn induces self-blame, particularly if someone believes they had control over the situation [43]. It is not surprising therefore that single women in this study were more likely to exhibit self-blame tendencies given they attributed their BV to their sexual behaviour choices—something they had control over. Conversely, for a number of women, knowing BV “was not an STI” offered a great sense of comfort and relief, most likely because they were able to mentally disassociate themselves from being “the type of woman that gets an STI” and shift blame for acquisition away from themselves [43]. Similar to our study, past research has also found that women in committed relationships often experience less self-blame following an STI diagnosis as their relationship and support of their partners provided a protective affect against moral judgment and feelings of shame and self-blame [46, 49, 50]. It was not surprising that WSW in this study experienced even higher levels of partner support particularly if their partner had experienced BV themselves.

### Strengths & Limitations

The strength of this study is that it is the first study we are aware of to explore in depth women’s views and experiences of the triggers of BV onset and factors associated with recurrence. We are not aware of any other study that has specifically explored this area in detail. A diverse range of women from varying sexual orientations, ethnic backgrounds, ages and relationship status’ were included in the study allowing for a rich, in-depth exploration of their views and experiences of recurrent BV. In offering the option of either telephone or face to face interviews, we were able to include a wider variety of participants due to greater flexibility in interview times and the privacy of telephone interviews. Interestingly women interviewed by telephone were often freer in discussing their experiences than women interviewed face to face, most likely due to the personal and intimate nature of the topic.

While a diverse range of women were purposively sampled a limitation of this sampling technique is the findings of the study may not be generalizable to broader populations of women from different geographic locations or cultural backgrounds. Future large scale studies should aim to explore the findings of this study to determine generalisability. A further limitation of this study is that women were not required to show evidence of their past BV diagnosis and therefore we cannot be certain all women had been diagnosed twice or more by a medical practitioner in the past five years. In stating this, the majority of women reported experiencing three or more past episodes of BV even if only professionally diagnosed twice.

### Future implications

In the face of unknown aetiology, increasing evidence of likely sexual transmission, high rates of recurrence and significant associated psychosocial sequelae [15], it is clear we need further basic research into the cause of BV and to look towards new strategies aimed at reducing BV recurrence and associated sequelae. Sufficiently powered male partner treatment trials are currently underway to determine the effectiveness of male partner treatment regimens. While the design of placebo-controlled female partner treatment trials may be ethically and practically more challenging, they are clearly needed and may hold the key to better understanding the nature and transmissibility of BV. The spectrum of bacteria involved in BV infection however will make the choice of agents to be used in these interventions challenging. Given the considerable BV related physical and psychosocial sequelae women experience, it is likely they will welcome new treatment approaches and management strategies if it may potentially offer a cure for BV.
